# Chemical composition, anti-toxoplasma, cytotoxicity, antioxidant, and anti-inflammatory potentials of *Cola gigantea* seed oil

**DOI:** 10.1080/13880209.2019.1577468

**Published:** 2019-03-24

**Authors:** O. Atolani, H. Oguntoye, E. T. Areh, O. S. Adeyemi, L. Kambizi

**Affiliations:** a Department of Chemistry, University of Ilorin, Ilorin, Nigeria;; b Medicinal Biochemistry, Nanomedicine and Toxicology Laboratory, Department of Biochemistry, Landmark University, Omu-Aran, Nigeria;; c Department of Horticulture, Cape Peninsula University of Technology, South Africa

**Keywords:** Fatty acid, *Toxoplasma gondii*, fibroblast, sterolic acid, stigmasterol, β-sitosterol, DPPH, ABTS, cell membrane stabilization

## Abstract

**Context:** *Cola gigantea* A. Chev. (Sterculiaceae) is an important medicinal tropical flora.

**Objective:** The seed oil of *C. gigantea*, an underutilized tropical plant was investigated for its antioxidant, anti-inflammatory, anti-*Toxoplasma,* and cytotoxicity activities as well as the chemical composition.

**Materials and methods:** The physicochemical parameters of the seed oil obtained via Soxhlet extraction was determined while the fatty acid and non-fatty acid component were analyzed by gas chromatography-mass spectrometry. The antioxidant activity was evaluated using 2,2-diphenyl-1-picrylhydrazyl (DPPH) and 2,2-azino-bis-(3-ethylbenzothiazoline-6-sulphonic acid) (ABTS) assays (10–50 µg/mL) while the anti-inflammatory property was determined through Cell Membrane Stabilization assay. The anti-parasite and cytotoxicity activity were evaluated (0–1000 µg/mL) using *Toxoplasma gondii* and mammalian cell line assays, respectively.

**Results:** The oil had fatty acids which ranged from C-12 to C-23 with linoleic (18:2) and palmitic acids (16:0) being dominant. The oil had 89.41% unsaturated fatty acids with sterolic acid, an uncommon acetylenic fatty acid reported for the first time. Non-fatty acids obtained include cholesterol (2.12%), campesterol (14.12%), stigmasterol (34.07%) and β-sitosterol (49.68%). The oil had a significantly (*p* < 0.05) low scavenging activity against DPPH radicals (IC_50_ > 50 µg/mL) compared with ascorbic acid. In contrast, the oil showed better activity against ABTS radicals (IC_50_ 44.19 ± 6.27 µg/mL) compared with ascorbic acid or quercetin. Furthermore, the oil showed anti-*T. gondii* and dose-dependent cytotoxicity in HFF cells with selectivity index (IC_50_/EC_50_ < 1).

**Discussion and conclusions:** The antioxidant potential of the oil suggests that it may serve as a potential source for various preparations for pharmaceuticals and cosmeceuticals.

## Introduction

*Cola gigantea* A. Chev. (Sterculiaceae), commonly known as giant Cola, is found mostly in relatively dry parts of the rain forest of the West Africa and West Indies (Olorode, 1984). The mature fruit of *Cola* species is a nut known as Kolanut (Duke 2001). It is an ever-green moderately sized tree often growing to a height of 25 m with glossy ovoid leaves up to 25 cm. Extracts of the plant are reported to have significant antimicrobial activity against a series of bacteria and fungi (Adeniyi et al. 2004; Reid et al. 2005; Sonibare et al. 2009; Agyare et al. 2012; Onyema and Ajiwe 2014). The leaf extracts are also acclaimed to boost blood supply, cure anaemia, sores, skin infection, and other inflammatory conditions. The nuts have a bitter flavour and high caffeine content (Benjamin et al. 1991; Blades 2000) and are often used for treatment of whooping cough, asthma, malaria, and fever (Odugbemi 2006). Additionally, the plant has other traditional relevance among which are the uses for increasing the capacity for physical exertion and enduring fatigue without food, stimulation of weak heart, and treating nervous debility, lack of emotion, depression, despondency, brooding, anxiety, and sea-sickness (Agyare et al. 2012). Phytochemical screening of the leaf extract indicated the presence of alkaloids, saponins, tannins, anthraquinones, and cardenolides (Sonibare et al. 2009). These phytochemicals might be responsible for the observed medicinal properties of the plant.

However, the chemical composition and corresponding biological profiling of the seed of *C. gigantea* have not been explored. This study reports for the first time the chemical composition, physicochemical properties, antioxidant, anti-inflammatory, anti-parasite and potential of the seed oil of *C. gigantea*.

## Materials and methods

### Plant material

*C. gigantea* seeds were obtained during winter of 2012 in Ogun State, Nigeria. The sample was identified by a renowned botanist, Mr. Bolu Ajayi, at the Herbarium of the Biological Sciences Department of the University of Ilorin, Ilorin, Nigeria. The seeds were removed from the fruit of *C. gigantea*, dried at room temperature, and pulverized. Pulverized seed material (710 g) was subjected to Soxhlet extraction using hexane for 3 h and the extract concentrated to obtain the oil.

### Preparation of sterol constituents for analysis

The oil (100 mg) was extracted with 10 mL saponification reagent [ethanol:33% KOH (w/v):20% ascorbic acid (94:6:0.5)]. 5α-Cholestane (100 μL) at a final concentration of 10 ppm was added as internal standard. The mixture was vortexed and subsequently incubated for 60 min at 50 °C. After the sample was cooled in ice water for 10 min, 5 mL deionized water and 5 mL hexane were added and the sample was vortexed and left to allow for separation into phases. The supernatant phase (1000 µL) was then dried in a speedvac. The dry samples were reconstituted with 200 µL dichloromethane and derivatized with 200 µL pyridine followed with 100 µL BSTFA and 1% TCMS. The mixture was vortexed and incubated for 1 h in an oven maintained at 50 °C. After incubation, the mixture was again vortexed and then transferred into a vial with an insert.

### Preparation of fatty acid methyl esters (FAMEs)

The oil was subjected to trans-esterification by treating 2 g of the oil with 10 mL 0.2 M methanolic H_2_SO_4_ following standard procedure (Atolani et al. 2016). The mixture was refluxed for an hour and then allowed to settle down. The organic phase containing the fatty acid methyl esters (FAMEs) was obtained using the separating funnel and thereafter concentrated via distillation. The FAMEs obtained was dried over anhydrous magnesium sulfate and kept for Gas Chromatography-Mass Spectrometry (GC-MS) analysis.

### Determination of saponification value

Saponification value was determined as described elsewhere (Fakhri and Qadri 2011; Atolani et al. 2016). Briefly, an aliquot of the oil (0.2 g) was taken in a conical flask and mixed with 25 mL methanolic sodium hydroxide (NaOH) plus three (3) drops of phenolphthalein indicator. The solution was thoroughly mixed, cooled in an ice bath and then titrated against 0.5 M HCl until the pink colour disappeared. The saponification value was thereafter estimated using the equation:

$$\text{SV}=\frac{\left(\text{Volume}\;\text{of}\;\left(S-B\right)\;\times M\;\times \;56.1\frac{g}{\text{mol}}\right)}{\text{Weight}\;\text{of}\;\text{sample}\;(g)}\;=\;\frac{\text{mgKOH}}{g}$$

where B = blank titer value (mL); S = sample titer value (mL); M = Molarity of KOH.

### Determination of acid value

Oil (0.2 g) was dissolved in 25 mL methanol and the mixture warmed in a water bath for 5 min with occasional shaking. Three drops of phenolphthalein indicator were added and the solution titrated against with 0.1 M NaOH, until the pink colouration disappears. The same procedure was followed for blank but without the sample (Atolani et al. 2016). The acid value was thereafter estimated using the expression:

$$\text{AV}\;=\;\frac{\text{Volume}\;\text{of}\;(\text{KOH})\;\times \;N\;\times \;56.1\;g/\text{mol}}{\text{Weight}\;\text{of}\;\text{sample}\;(g)}\;=\frac{\text{mgKOH}}{g}$$

where AV = Acid value; M = Molarity of KOH

### Antioxidant activities

The antioxidant capacity of the oil was established by using two complimentary antioxidant assays; the 2,2-diphenyl-1-picrylhydrazyl (DPPH) and 2,2 azino-bis-(3ethylbenzothiazoline-6-sulphonic acid) (ABTS) scavenging assays.

### 2, 2-Azinobis-3-ethylbenzothiazoline-6-sulfonate (ABTS) scavenging assay

The ABTS assay was carried out as described previously (Atolani et al. 2013; 2018; Kambizi et al. 2017). Briefly, 7 mM ABTS in deionized water was mixed at ratio 1:1 with freshly prepared potassium persulfate (2.45 mM) and the mixture kept in the dark for 24–48 h. The ABTS solution was diluted in methanol at 1:25. Aliquot (20 μL) of samples (10–50 µg/mL) was added to 2 mL of ABTS^+^ solution, and the mixture kept at a standard temperature of 30 °C. The absorbance was measured at 734 nm at 10 min after initial mixing. All analysis was determined in triplicate. The % ABTS scavenging activity was calculated against the control by using the expression:

$$\text{Antioxidant Capacity},\text{ AOC}=[\left({\text{A}}_{\text{control}}–{\text{A}}_{\text{sample}}\right)\times \text{100}]/({\text{A}}_{\text{control}})$$

where A_control_ and A_sample_ represent the absorbances of the control and the samples, respectively.

### 2, 2-Diphenyl-1-picrylhydrazyl (DPPH) Free radical scavenging assay

The DPPH assay was carried out following a previously described protocol (Atolani et al. 2013; 2015). The DPPH reagent was freshly prepared and kept in a dark bottle in the refrigerator overnight. The standard antioxidant (ascorbic acid) and extracts were prepared in triplicate at between 10–50 µg/mL. Aliquot (1 mL) of DPPH solution was added to all samples and this followed immediately by incubation in the dark for 30 min. The absorbance value was measured at 517 nm and the % antioxidant activity was estimated against the control using the expression:

$$\text{Antioxidant}\;\text{Activities},\;\text{AA}=[\left(A_{\text{control}}–A_{\text{sample}}\right)\times 100]/\left(A_{\text{contro}}l\right)$$

where A_control_ and A_sample_ represent the absorbances of the control and the samples, respectively.

### Anti-Inflammatory (red blood cell membrane stabilization) Assay

The procedure described previously for estimating anti-inflammatory potential (Kambizi et al. 2017) was adopted. Mice were kept for 2 weeks without any contact with nonsteroidal anti-inflammatory drugs. Thereafter, blood samples were collected from the mice mixed with equal volume of Alsever solution (2% dextrose, 0.8% sodium citrate, 0.5% citric acid and 0.42% NaCl). The mixture was subjected to centrifugation at 3000 rpm to obtain packed cells which were washed with isosaline solution and a 10% portion of the suspension was reconstituted with normal saline. Hyposaline solution (2 mL), 0.5 mL RBC suspension and 1 mL phosphate buffer was added to the extracts or diclofenac (reference drug) prepared in triplicate in distilled water at different concentrations (50 and 100 µg/mL). The mixture was incubated at 37 °C for 30 min and thereafter the mixture was centrifuged at 3000 rpm for 20 min. Then the absorbance was recorded at 560 nm and the extent of the stabilization was estimated by using the formula:

$$\text{Stabilization}\left(\%\right)=\text{1}00-({\text{A}}_{\text{t}}/{\text{A}}_{\text{c}})\times 100$$

where A_t_ and A_c_ are the respective absorbance of the test samples and the control.

### *In vitro* anti-parasite assay

The *T. gondii* RH strain 2 F (ATCC^®^ 50839) was used for the anti-parasite study. The parasite was maintained by repeated passages in HFF cells cultured in DMEM (Nissui, Tokyo, Japan) and supplemented with GlutaMAX™-I (Gibco, Invitrogen, UK), 10% (v/v) fetal calf serum (FCS; Gibco, Invitrogen, UK), and penicillin and streptomycin (100 U/mL; Biowhittaker, UK). HFF cells infected with *T. gondii* tachyzoites were passed through a 27-gauge needle to lyse them. The cell lysates were then filtered through a 5 µm filter to obtain a tachyzoite suspension free of host cell debris. The parasite suspension was washed with fresh culture medium and the *in vitro* parasite growth inhibition assays were performed as previously described (Adeyemi et al. 2017). Briefly, the oil extracts (reconstituted in culture medium) were incubated with freshly lysed and purified parasites in growing HFF cells in 96-well optical bottom plates (Nunc; Fisher Scientific, Pittsburgh, USA). Medium-treated cells served as negative drug control. Sulfadiazine was included a positive drug control. After 72 h incubation at 37 °C in a 5% CO_2_ atmosphere, the viability of the RH-2F parasite strain was determined by assaying for galactosidase activity by using a B-Glo luminescent assay kit (Promega, Madison, USA). The assay was performed in triplicate and repeated three times.

### Measurement of intracellular reactive oxygen species (ROS)

Measurement of intracellular ROS was as previously described (Adeyemi et al. 2017). This is premised on the intracellular peroxide-dependent oxidation of 2′,7′-dichlorodihydro-fluorescein diacetate (H_2_DCF-DA, Sigma, St. Louis, MO, USA) to form the fluorescent compound 2′,7′-dichlorofluorescein (DCF). Briefly, growing HFF monolayers were treated with *C. gigantea* in the absence/presence of *T. gondii* infection. After 24 h incubation at 37 °C and 5% CO_2_ atmosphere, the cells were harvested, purified, and re-suspended in PBS containing the H_2_DCF-DA to a final concentration of 100 µM. The cell suspension containing the fluorescent dye was incubated for 30–60 min at 37 °C. Fluorescence acquisition was recorded by using a spectrofluorometer (Corona Electric, Japan) with excitation set at 485 nm and emission at 530 nm. H_2_O_2_ was included as a positive control in order to validate the ROS detection assay.

### Measurement of the mitochondrial membrane potential (MMP)

The measurement of MMP was as described elsewhere (Adeyemi et al. 2017). Briefly, growing HFF cells were treated with *C. gigantea* oil in the absence/presence of *T. gondii* infection. After 24 h incubation at 37 °C and 5% CO_2_ atmosphere, the cells were harvested, purified, and stained with 200 nM MitoRed (Dojindo Molecular Technologies Inc. Japan) by following the manufacturer’s protocol. Fluorescence measurement was acquired by using a spectrofluorometer (Corona Electric, Japan) with excitation set at 560 nm and emission at 580 nm.

### Cytotoxicity of the oil in mammalian cell

Human foreskin fibroblast (HFF, ATCC^®^) cells were maintained in normal cell culture medium (DMEM, GlutaMAX™-I, 10% (v/v) fetal calf serum and penicillin and streptomycin. Confluent cells were harvested as per sub-culture protocol and re-suspended to the desired cell density. The cells were seeded onto 96-well plates (Nunc; Fisher Scientific, Pittsburgh, USA) at a density of 1 × 10^5^ cells per well and incubated for 72 h followed by treatment with various concentrations (0–1000 µg/mL) of the oil extracts. Culture medium lacking the test compounds was added to the control well Staurosporine was included as positive drug control. After 72 h incubation at 37 °C and 5% CO_2_ atmosphere, the cell viability was determined by using the CellTitre-Aqueous One Solution proliferation assay kit (Promega, Madison, USA) and following the manufacturer’s instruction. The absorbance signal was recorded at 490 nm by using a microplate reader (MTP 500; Corona Electric, Japan). The assay was in triplicates and repeated three times independently.

### GC-MS analysis of sterol constituents

Chromatographic separation of the compounds was performed on a gas chromatograph. Aliquot (1 µL) of the sample was injected into an Agilent 6890 N (Agilent, Palo Alto, CA) coupled to an Agilent 5975 MS mass spectrometer detector, using a Zebron AB-MultiResidue II (30 by 0.25 mm ID, 0.25 µm film thickness) column (Part No. 7HG-G016-11). The oven temperature program was maintained at 100 °C for 2 min, ramped at 15 °C/min to 180 °C held for 0 min, ramped at 5 °C/min to 250 and held for 3 min and finally at 20 °C/min to 320 °C held for 12 min. The total run time was 40 min. The carrier gas was helium at a flow rate of 1.2 mL/min and the injector temperature was maintained at 200 °C and operated in a splitless mode. The Mass spectral data were recorded on a MSD operated in full scan mode (35–600 *m/z*) with both the ion source and quadruple temperatures maintained at 240 °C and 150 °C, respectively. The transfer line temperature was maintained at 200 °C. Solvent Delay was held at 5.00 min. The compounds were identified on the basis of fragment pattern obtained and comparison of the retention time with that of authentic samples injected.

### GC-MS analysis of FAMEs

The FAMEs obtained from the seed oil of *C. gigantea* were analyzed on gas chromatograph GC QP2010SE SHIMADZU, Japan, equipped with a silica capillary HP-5MS column (30 m by 0.32, 0.5 µm) coupled directly to a mass detector (Agilent Technology MSD). Helium served as the carrier gas. The injector was operated at 250 °C and while the detector was operated at 380 °C. The oven temperature was programmed to rise from 40 to 250 °C at a heating rate of 5 °C/min. The analyses were performed using a split mode (1:10). Scan start time-end time; 3.77–29.31 min. The instrument was calibrated using authentic samples of n-alkanes for the reliability of characterization of compounds. The mass range of the mass spectrometer was set to 45–650 m/z. The FAMEs were identified on the basis of the authentic samples previously injected in combination with the examination with individual molecular weight, mass spectra and comparison of fragmentation pattern in the mass spectrum with that of the National Institute of Science and Technology, NIST library.

### Data analysis

Results were analyzed by using a one-way ANOVA (GraphPad Software Inc., San Diego, CA, USA) and data are presented as the mean ± standard error of mean (SEM) except otherwise indicated. All experiments were performed in triplicate. The concentration of the oil showing a 50% inhibition or reduction in parasite and/or cell viability (i.e., IC_50_ values) were estimated from a dose-response curve while a non-linear regression analysis was used to fit the curve. Values at *p* < 0.05 were taken as significant.

## Results and discussion

### Physicochemical characterization

The physico-chemical characteristics of the seed oil of *C. gigantea* are shown in Table 1. The oil had saponification value of 45.623 mg KOH/g. This value was considerably low considering its potential for industrial purposes (such as soap manufacture) and the suitability for human consumption. According to Codex Standards for Edible Fats and Oils, the maximum level of acid value permitted for refined fats and oils is 0.6 mg KOH/g fat or oil. The acid value recorded for *C*. *gigantea* in this study is 0.25 mg/g. The acid value of the oil is within a range acceptable for edible purposes. The low acid value of oil is an indication for stability over a long period of time. The low acid value of the oil also reveals that the oil would be less susceptible to rancidity.

**Table 1.** The physiochemical characteristics of the oil.

Parameters	Value
Colours	Yellow
% yield of soxhlet oil (w/w)	≈1.0
% yield of the trans-esterified oil (w/w)	30.37
Saponification value (mgKOH/g)	45.63
Acid value (mgKOH/g)	0.2 5

### Antioxidant activities

The antioxidant activities of the oil were examined using the DPPH and ABTS assays. The extent of ABTS radical scavenging activity of the oil compared to the reference compound, quercetin is as depicted (Supplementary data 1). The oil had a dose-response ABTS radical scavenging activity with highest activity observed at the highest concentration (50 µg/mL) tested. ABTS radical scavenging activity (Table 2) of *C. gigantea* oil (IC_50_ = 44.19 ± 6.27 µg/mL) indicated that the oil has good antioxidant potential although not significant (at *p* < 0.05) when compared to quercetin (IC_50_ = 75.14 ± 4.32 µg/mL).

**Table 2.** IC_50_ values of the radical scavenging activity of the seed oil.

IC_50_ (µg/mL)	Ascorbic acid	Quercertin	*C. gigantea* seed oil
ABTS	75.15 ± 4.32	76.71 ± 0.40	44.19 ± 6.27
DPPH	13.55 ± 0.56	ND	≥50*

ND: Not determined. Data are expressed as Mean ± Standard Error of Mean of triplicate determinations. *Values are Significant at *p* < 0.05.

The percentage DPPH radical scavenging activity of *C. gigantea* seed oil, examined by dose-response, was lower than the reference compound, ascorbic acid (Supplementary data 2). The highest activity of 44.52% was recorded at the highest concentration, 50 µg/mL meaning the IC_50_ value was >50 µg/mL (Table 2). This suggests that the oil had low scavenging activity for DPPH radical compared with ascorbic acid.

### Anti-inflammatory potential

**Figure 1.**
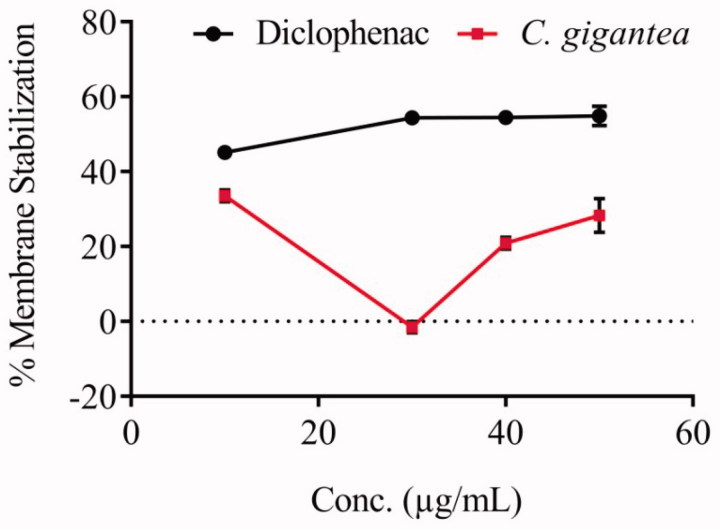
Membrane stabilization potential of *C. gigantea* seed oil.

The anti-inflammatory activity of *C. gigantea* seed oil was evaluated through cell membrane stabilization assay. The oil does not clearly exhibit a dose-response activity as compared to the standard, diclofenac (Figure 1). The IC_50_ value of the oil (0.23 ± 3.30 µg/mL) reveals that the oil exhibited good anti-inflammatory property in membrane stabilization of red blood cell.

### Fatty acids profile of *C. gigantea* seed oil

The GC-MS analysis of the trans-esterified seed oil of the *C. gigantea* revealed the presence of nine fatty acids (Table 3). The major fatty acids are linoleic acid (40.67%), palmitic acid (20.26%), 9,12-hexadecanoic acid (13.45%), stearolic acid (11.69%) and stearic acid (9.33%). Other fatty acids were obtained in minute quantities. The oil had 89.41% unsaturated fatty acids with sterolic acid accounting for 13.07% of the total unsaturated content. The stearolic acid (18:2) also known as 9-octadecynoic acid is an uncommon acetylenic fatty acid suggested to be an important fatty acid with novel DNA binding capacity (Thomasson 1954; Berry et al. 1991). It is reported to be present in the seeds of Olacaceae and Santalaceae families (Okada et al. 2013). An odd number fatty acid (10-nonadecenoic acid, 19:1) was obtained in small quantity. Linoleic (18:2) and palmitic acids (16:0) were the dominant and sub-dominant fatty acids in the oil, implying that C*. gigantea* can be a good source of edible.

**Table 3.** Fatty acids composition of C*. gigantea* seed oil.

S/N	RT (Min)	Name of Compound	Mol. Formula	% Composition
1	8.84	Lauric acid	C_12_H_24_O_2_	0.65
2	10.72	Myristic acid	C_14_H_28_O_2_	0.39
3	12.95	Palmitic acid	C_16_H_32_O_2_	20.26
4	13.49	Stearolic acid	C_18_H_32_O_2_	11.69
5	13.86	9,12-Hexadecanoic acid	C_16_H_28_O_2_	13.45
6	14.11	Linoleic acid	C_18_H_32_O_2_	40.67
7	14.33	Stearic acid	C_18_H_36_O_2_	9.33
8	15.04	10-Nonadecenoic acid	C_19_H_36_O_2_	3.34
9	17.50	Tricosanoic acid	C_23_H_46_O_2_	0.22
			Total Saturates	10.59
			Total Unsaturates	89.41

### Sterol composition of the *C. gigantea* seed oil

The sterol composition in the seed oil of *C. gigantea* was determined following standard procedure. The quantitative GC-MS results indicated the presence of four sterols which include cholesterol, campesterol, stigmasterol and β-sitosterol at 2.12, 14.12, 34.07 and 49.68%, respectively (Table 4 and Supplementary data 3). β-Sitosterol was apparently the most abundant sterol in the seed.

**Table 4.** Chemical composition of the non-fatty acids constituent of *C. gigantea* seed oil.

S/N	Retention Time (Min)	Sterol	Relative Abundance (%)
1	28.82	Cholesterol	2.12
2	29.54	Campesterol	14.12
3	29.76	Stigmasterol	34.07
4	30.26	Beta-Sitosterol	49.68

### *In vitro* anti-parasite potential

**Figure 2.**
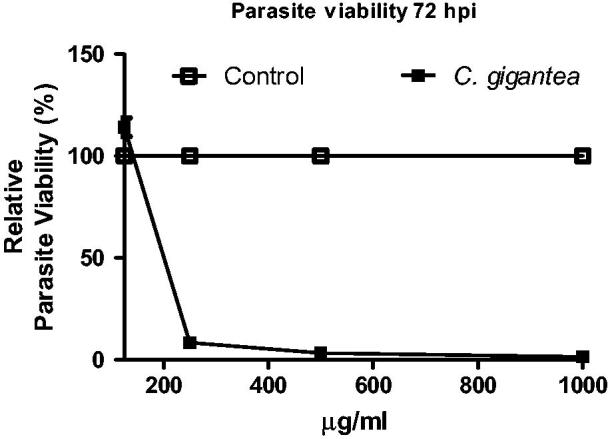
Anti-*Toxoplasma gondii* activity of *C. gigantea* oil.

The *in vitro* anti-parasite assay was carried out in order to evaluate the potency of *C gigantea* oil to inhibit the parasite growth using the *T. gondii* as a proof-of-principle. The oil showed dose-dependent action against *T. gondii* (Figure 2); the lowest concentration had the highest cell viability. The anti-parasite of *C gigantea* is significantly high when compared with the negative drug control thus suggesting that *C gigantea* seed oil can be explored as alternative anti-parasite agent.

### Addition of Trolox failed to abate the anti-parasitic activity of *C. gigantea* oil

**Figure 3.**
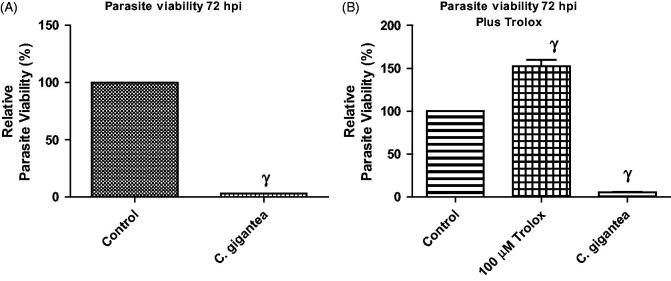
(A,B) Anti-*T. gondii* activity of *C. gigantea* in the absence/presence of α-tocopherol (Trolox). Values are expressed as the Mean ± SEM (*n* = 3). Each experiment was in triplicates and performed three times independently. γ is significant at *p* < 0.0001 versus control.

The reactive oxygen species (ROS) was determined in order to show whether ROS was culpable in the anti-parasite action of the *C. gigantea* oil. An antioxidant, Trolox (100 µM) was included in the screening assay and data showed that the addition of Trolox failed to relieve the parasite growth restriction capability of the *C gigantea* (Figure 3(A,B)). This suggests that oxidative stress might not be contributing to the anti-parasite action of the oil extract.

**Figure 4.**
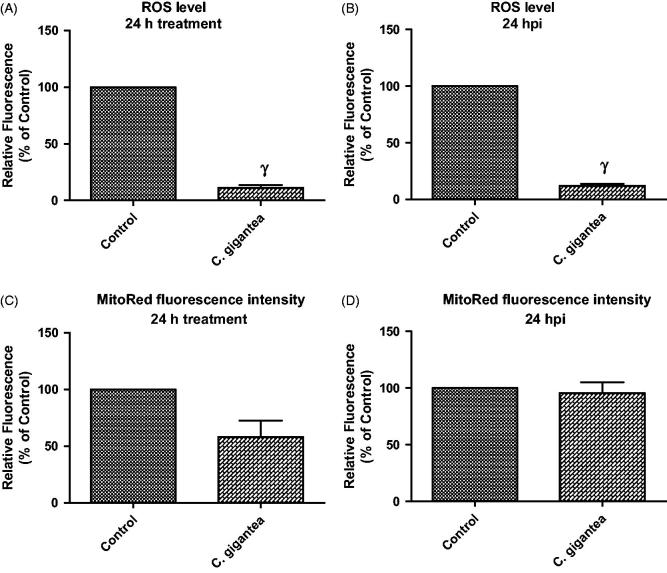
ROS level and mitoRed fluorescence intensity following 24 h treatment with *C. gigantea*; (A) In the absence of *T. gondii* infection; (B) In the presence of *T. gondii* infection; (C) In the absence of *T. gondii* infection; (D) In the presence of *T. gondii* infection. Values are expressed as the Mean ± SEM (*n* = 3). Each experiment was done in triplicates and performed three times independently. γ is significant at *p* < 0.0001 versus control.

Probably, *C. gigantea* did not predispose to the generation of ROS but utilizes alternative ways to restrict parasite growth. Moreover, data revealed that the *C. gigantea* oil extract actually reduced the ROS level by ≥70% when compared with the negative drug control (Figure 4(A,B)). This is thus suggesting that the *C. gigantea* oil extract might possess some level of antioxidant capacity and further confirmed the *in vitro* antioxidant potential as determined in this study. In contrast, we found that the *C. gigantea* oil extract mildly affected the cellular MMP (Figure 4(C)) but not in the presence of *T. gondii* infection (Figure 4(D)). The reason for this is unknown but may further support the fact the anti-parasite action of the *C. gigantea* oil extract precludes oxidative stress. Consequently, the *C. gigantea* oil also reduced cellular mitochondria membrane potential (MMP) in the absence of the *T. gondii* infection (Figure 4(C)), but not in the presence of the *T. gondii* infection (Figure 4(D)). This probably might be due to alteration of physiological status of cells due to *T. gondii* infection.

### Cytotoxicity of oil extracts in mammalian cells

**Figure 5.**
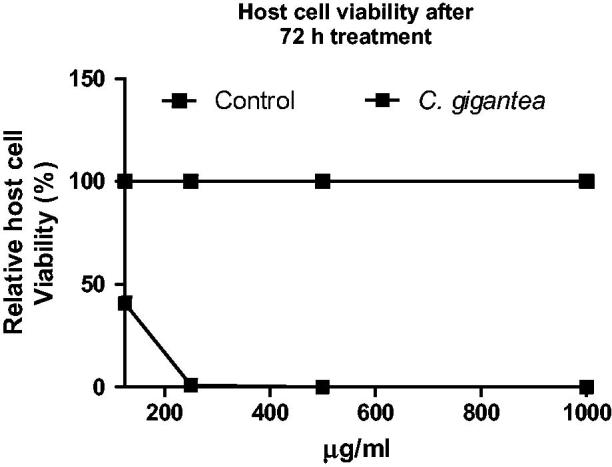
Cell viability of *C. gigantea* oil using HFF monolayers. Cell viability values were calculated relative to background values (0% viability) and the untreated negative control (100% viability). Staurosporine (1 µM) was included as positive control to validate the assay. Values are expressed as the Mean ± SEM (*n* = 3). Each experiment was in triplicates and performed three times independently.

Here, the cytotoxic action of the *C. gigantea* in HFF cells was determined. Data showed that the *C. gigantea* oil extract exhibited dose-dependent cytotoxic action against the HFF monolayers (Figure 5).

### Selectivity index (SI)

In order to determine the anti-parasitic efficacy of the *C. gigantea* oil, we estimated the SI of the oil which is the ratio of the cytotoxicity (IC_50_) in host cells to the anti-parasite activity (EC_50_). Data revealed that *C. gigantea* had SI of ≤1 relative to the reference drug, sulfadiazine, (SI ≤ 4), the drug currently used for treatment of toxoplasmosis (Table 5). *C. gigantea* oil failed to show promising specific anti-parasitic activity which may indicate that the anti-parasitic activity of it might be due to general cellular toxicity.

**Table 5.** Selectivity Index of *C. gigantea* seed oil.

Sample	Anti-parasite activity EC_50_ (µg/mL)	Host cell cytotoxicity IC_50_ (µg/mL)	Selectivity index: IC_50_/EC_50_
*C. gigantea*	≤15	≤10	<1*
Sulfadiazine	≤150	≤500	<4

ND: Not determined. Data are expressed as Mean ± Standard Error of Mean of triplicate determinations. *Values are Significant at *p* < 0.05.

## Conclusions

The chemical constituent of *C. gigantea* seed has been investigated. The GC-MS analysis revealed the presence of 12 fatty acids of which palmitic and stearolic acids were the dominant fatty acids. Four sterols, namely cholesterol, campesterol, stigmasterol, and β-sitosterol were detected and quantified. Our data showed that the *C. gigantea* oil extract possesses mild antioxidant as well as an anti-inflammatory potential. Further, data suggest that the anti-parasite action of the *C. gigantea* oil extract might be as a result of general cellular toxicity and that this toxic effect precludes ROS production and/or oxidative stress. Overall, data shows the bio-activity potential of the oil from the underutilized seed has significant capacity for application in foods and cosmetics. The potential in the seed could be cheaply harnessed for future chemical and biochemical applications.

## Supplementary Material

Supplementary_data_C_Gigantea.docx
