# Supplementary material for: Chemical composition, anti-toxoplasma, cytotoxicity, antioxidant, and anti-inflammatory potentials of Cola gigantea seed oil
Source: Pharm Biol. 2019 Mar 24;57(1):154–60. doi: 10.1080/13880209.2019.1577468 (PMC6442224; doi:10.1080/13880209.2019.1577468)
Supplement: Supplementary_data_C_Gigantea.docx [file IPHB_A_1577468_SM6236.docx]

**Supplementary data 1:** ABTS Radical Scavenging Activities of *C*. *gigantea* Oil Compared with Quercertin

**Supplementary data 2:** DPPH Radical Scavenging Activity of *C. gigantea* seed oil


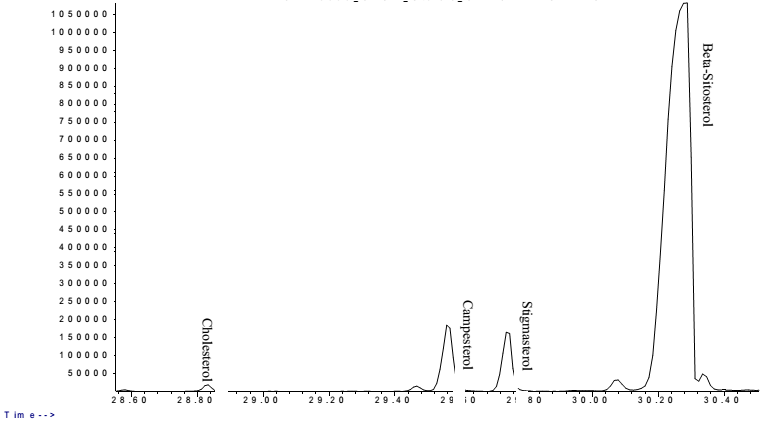


**Supplementary data 3:** Total Ion Chromatogram of the sterol constituent of *C. gigantea* seed oil
